# Supplementary material for: Association of serum 25-hydroxyvitamin D levels with severe necroinflammatory activity and inflammatory cytokine production in type I autoimmune hepatitis
Source: PLoS One. 2020 Nov 5;15(11):e0239481. doi: 10.1371/journal.pone.0239481 (PMC7643962; doi:10.1371/journal.pone.0239481)
Supplement: S1 Table — (DOCX) [file pone.0239481.s001.docx]

**Supporting TABLE 1.** Characteristics of patients with AIH, DILI, acute hepatitis B, and healthy individuals

|  | HC  (n=10) | AIH  (n=66) | Acute  AIH  (n=34) | DILI  (n=11) | Acute  hepatitis B  (n=3) |
| --- | --- | --- | --- | --- | --- |
| Age, years,  median (IQR) | 55  (49-59) | 57  (50-66) | 57  (47-66) | 54  (44-65) | 52  (49-64) |
| Sex, male/female  (female %) | 1/9  (90%) | 7/59  (89%) | 5/29  (85%) | 3/8  (73%) | 1/2  (67%) |
| Laboratory data |  |  |  |  |  |
| PLT, ×10^4^/µL,  median (IQR) | NA | 16  (13-21) | 16  (13-23) | 19  (16-26) | 27  (16-32) |
| ALB, g/dL,  median (IQR) | NA | 3.5  (3.0-3.8) | 3.4  (2.9-3.8) | 3.4  (3.2-3.8) | 3.5  (2.9-3.9) |
| TB, mg/dL,  median (IQR) | NA | 2.0  (0.9-8.2) | 7.1  (1.9-16.1) | 6.5  (3.3-9.9) | 1.8  (1.4-7.9) |
| PT, %, median  (IQR) | NA | 74  (56-89) | 62*  (47-86) | 85*  (70-105) | 82  (64-91) |
| ALT, U/L,  median (IQR) | NA | 247  (98-815) | 799  (371-1107) | 350  (283-1037) | 865  (649-1029) |
| ALP, U/L,  median (IQR) | NA | 399  (299-544) | 410  (346-544) | 564  (474-765) | 784  (620-819) |
| IgG, mg/dL,  median (IQR) | NA | 2530  (1911-3171) | 2315*  (1776-3015) | 1405*  (1094-1473) | 1780  (1420-2270) |
| Total 25(OH) D,  ng/mL (IQR) | 18.2^¶^  (14.4-23.6) | 14.2^¶^  (11.4-17.9) | 13.2  (10.2-16.0) | 8.8  (7.9-11.9) | 14.4  (9.2-16.4) |

**P* < 0.05 was considered significant [Acute AIH vs. DILI].

^¶^*P* < 0.05 was considered significant [HC vs. AIH].

Abbreviations: AIH, autoimmune hepatictis; ALB, albumin; ALP, alkaline phosphatase; ALT, alanine aminotransferase; DILI, drug-induced liver injury; HC, healthy control; IgG, immunoglobulin G; IQR, interquartile range; NA, not available; PLT, platelet count; PT, Prothrombin time; TB, total bilirubin; 25(OH)D, 25-hydroxyvitamin D.
