# Supplementary material for: Association of serum 25-hydroxyvitamin D levels with severe necroinflammatory activity and inflammatory cytokine production in type I autoimmune hepatitis
Source: PLoS One. 2020 Nov 5;15(11):e0239481. doi: 10.1371/journal.pone.0239481 (PMC7643962; doi:10.1371/journal.pone.0239481)
Supplement: S2 Table — (DOCX) [file pone.0239481.s002.docx]

**Supporting TABLE 2.** Relationship between total 25-hydroxyvitamin D and clinical presentation in patients with DILI

| Variable | Total 25(OH)D | |
| --- | --- | --- |
|  | r | *P* |
| ALT (U/L) | 0.2232 | 0.5071 |
| ALP (U/L) | -0.1412 | 0.6674 |
| TB (mg/dL) | -0.1002 | 0.7601 |
| ALB (g/dL) | 0.6324 | 0.0403* |
| PT (%) | 0.0820 | 0.8114 |
| PLT (x10^4^/μL) | -0.1822 | 0.5806 |
| IgG (mg/dL) | 0.1412 | 0.6783 |

**P* < 0.05 was considered significant.

Abbreviations: ALB, albumin; ALP, alkaline phosphatase; ALT, alanine aminotransferase; DILI, drug-induced liver injury; IgG, immunoglobulin G; PLT, platelet count; PT, Prothrombin time; TB, total bilirubin; 25(OH)D , 25-hydroxyvitamin D.
