# Supplementary material for: Association of serum 25-hydroxyvitamin D levels with severe necroinflammatory activity and inflammatory cytokine production in type I autoimmune hepatitis
Source: PLoS One. 2020 Nov 5;15(11):e0239481. doi: 10.1371/journal.pone.0239481 (PMC7643962; doi:10.1371/journal.pone.0239481)
Supplement: S3 Table — (DOCX) [file pone.0239481.s003.docx]

**Supporting TABLE 3.** Relationship between the percentage of free 25-hydroxyvitamin D and clinical presentation in patients with AIH

| Variable | Percent Free 25(OH)D | |
| --- | --- | --- |
|  | r | *P* |
| AST (U/L) | 0.2375 | 0.0631 |
| ALT (U/L) | 0.1605 | 0.1980 |
| ALP (U/L) | -0.05603 | 0.6550 |
| TB (mg/dL) | 0.4239 | 0.0004* |
| ALB (g/dL) | -0.3137 | 0.0146* |
| PT (%) | -0.3518 | 0.0044* |
| PLT (x10^4^/μL) | -0.1165 | 0.3592 |
| IgG (mg/dL) | 0.1151 | 0.3572 |
| Staging of fibrosis | 0.2500 | 0.0520 |
| Grading of activity | 0.4163 | 0.0008* |

**P* < 0.05 was considered significant.

Abbreviations: ALB, albumin; TB, total bilirubin; AST, aspartate aminotransferase; ALT, alanine aminotransferase; PLT, platelet count; PT, Prothrombin time; ALP, alkaline phosphatase; IgG, immunoglobulin G; Free 25(OH)D , free 25-hydroxyvitamin D.
