# Supplementary material for: Association of serum 25-hydroxyvitamin D levels with severe necroinflammatory activity and inflammatory cytokine production in type I autoimmune hepatitis
Source: PLoS One. 2020 Nov 5;15(11):e0239481. doi: 10.1371/journal.pone.0239481 (PMC7643962; doi:10.1371/journal.pone.0239481)
Supplement: S4 Table — (DOCX) [file pone.0239481.s004.docx]

**Supporting TABLE 4.** Relationship between the percentage of free 25-hydroxyvitamin D and cytokine profile in patients with AIH

| Variable | Percent Free 25(OH)D | |
| --- | --- | --- |
|  | r | *P* |
| IL-1β (pg/ml) | -0.2334 | 0.0593 |
| IL-4 (pg/ml) | 0.1539 | 0.2173 |
| IL-6 (pg/ml) | 0.1357 | 0.2774 |
| IL-10 (pg/ml) | 0.06689 | 0.5936 |
| IL-17A (pg/ml) | -0.1399 | 0.2625 |
| IL-17F (pg/ml) | 0.07958 | 0.5253 |
| IL-21 (pg/ml) | 0.1410 | 0.2589 |
| IL-22 (pg/ml) | -0.05165 | 0.6805 |
| IL-23 (pg/ml) | -0.2248 | 0.0695 |
| IL-25 (pg/ml) | NA | NA |
| IL-31 (pg/ml) | -0.1150 | 0.3580 |
| IL-33 (pg/ml) | -0.06130 | 0.6249 |
| IFN-γ(pg/ml) | 0.1121 | 0.3700 |
| TNF-α (pg/ml) | 0.01436 | 0.9089 |
| sCD40L (pg/ml) | -0.2628 | 0.0330* |

**P* < 0.05 was considered significant.

Abbreviations: IL, interleukin; IFN, interferon; TNF, tumor necrosis factor; Free 25(OH)D , free 25-hydroxyvitamin D; sCD40L, soluble CD40 ligand; NA, not available.
